# Defining the short-term effects of pharmacological 5′-AMP activated kinase modulators on mitochondrial polarization, morphology and heterogeneity

**DOI:** 10.7717/peerj.5469

**Published:** 2018-08-30

**Authors:** Mohamed Kodiha, Etienne Flamant, Yi Meng Wang, Ursula Stochaj

**Affiliations:** Department of Physiology, McGill University, Montreal, Canada

**Keywords:** Mitochondria, Mitotracker, 5′-AMP activated kinase, pharmacological AMPK modulator

## Abstract

**Background:**

Under aerobic growth conditions, mitochondria are the major producers of cellular ATP and crucial for the proper performance of organs and tissues. This applies especially to cells with high energy demand, such as the renal proximal tubule epithelium. Mitochondrial dysfunction contributes to the pathology of human health conditions, including various kidney diseases. The improvement of mitochondrial function ameliorates some of these pathologies. This can potentially be achieved with pharmacological compounds. For example, long-term treatment with activators of 5′-AMP activated kinase (AMPK) enhances mitochondrial biogenesis. However, pharmacological damage control during acute cell injury requires that the short-term effects of these compounds and the impact on healthy cells are also understood. It was our objective to define the changes elicited by established modulators of AMPK activity in healthy renal proximal tubule cells.

**Methods:**

Our work combines confocal microscopy with quantitative image analysis, 3D image reconstruction and Western blotting to provide novel insights into the biology of mitochondria. Specifically, we evaluated the effects of pharmacological AMPK modulators (compound C, AICAR, phenformin, resveratrol) on mitochondrial polarization, morphology and heterogeneity. Microscopic studies generated information at the single cell and subcellular levels. Our research focused on LLC-PK1 cells that are derived from the renal proximal tubule. Mitochondrial heterogeneity was also examined in MCF7 breast cancer cells.

**Results:**

Pharmacological agents that affect AMPK activity in renal proximal tubule cells can alter mitochondrial organization and the electrochemical potential across the inner mitochondrial membrane. These changes were compound-specific. Short-term incubation with the AMPK inhibitor compound C caused mitochondrial hyperpolarization. This was accompanied by mitochondrial fragmentation. By contrast, AMPK activators AICAR, phenformin and resveratrol had little impact. We further show that the biological properties of mitochondria are determined by their subcellular location. Mitochondria at the cell periphery displayed higher MitoTracker/Tom70 values as compared to organelles located in the vicinity of the nucleus. This was not limited to renal proximal tubule cells, but also observed in MCF7 cells. Pharmacological AMPK modulators altered these location-dependent properties in a compound-specific fashion. While the region-dependent differences were enhanced with phenformin, they were ameliorated by resveratrol.

**Discussion:**

We evaluated the rapid changes in mitochondrial characteristics that are induced by pharmacological AMPK modulators. Our research supports the concept that pharmacological agents that target AMPK can rearrange mitochondrial networks at the single cell level. Collectively, these insights are relevant to the development of proper strategies for the short-term adjustment of mitochondrial performance.

## Introduction

The kidney is one of the most energy-demanding organs in humans and characterized by a high metabolic rate (Bhargava & Schnellmann, 2017). Kidney cells contain numerous mitochondria to promote efficient ATP production via oxidative phosphorylation (Bhargava & Schnellmann, 2017; Szeto, 2017). Within the kidney, proximal tubules reabsorb various organic molecules and ions via active transport processes that require large energy supplies. This energy supply is provided predominantly by mitochondria that are particularly abundant in proximal tubule cells. The reliance on mitochondrial ATP production makes proximal tubules especially vulnerable to organelle dysfunction. As a result, kidney health is severely affected by mitochondrial impairment. For example, malfunctional mitochondria are implicated in acute kidney disease, diabetic renal nephropathy and sepsis (Bhargava & Schnellmann, 2017; Gomez, Jin & Kellum, 2015).

Different pharmacological approaches have been used to overcome the loss of proper mitochondrial performance in diseased and aging kidneys (Bhargava & Schnellmann, 2017; Decleves & Sharma, 2014; Gomez, Jin & Kellum, 2015). These include targeting of the ser/thr protein kinase 5′-AMP activated kinase (AMPK), a critical regulator of lipid, carbohydrate and protein homeostasis (Hardie, 2018; Trewin, Berry & Wojtovich, 2018). Due to its ability to activate catabolism while repressing anabolic pathways, AMPK is a key player for many human diseases and disorders (Coughlan et al., 2014; Fogarty & Hardie, 2010; Kodiha, Mahboubi & Stochaj, 2013; Kodiha & Stochaj, 2011a; Kodiha & Stochaj, 2011b; Viollet et al., 2010).

AMPK is a heterotrimeric enzyme (*αβγ*), composed of a catalytic *α* subunit as well as regulatory *β* and *γ* subunits (Hardie, Hawley & Scott, 2006). Phosphorylation of the *α* subunit on Thr183 or Thr172 (AMPK*α*1 or AMPK*α*2 subunit) activates AMPK when the cellular energy state decreases (Steinberg & Kemp, 2009). AMPK activation has acute and long-term physiological consequences (Mantovani & Roy, 2011). Long-term effects are mediated by changes in gene expression; a prominent example is *de novo* mitochondrial biogenesis (Hardie, Ross & Hawley, 2012). By contrast, the immediate outcomes are the phosphorylation of downstream AMPK targets, which rapidly downregulates ATP-consuming pathways and shifts the metabolic state towards catabolism.

AMPK can be activated in kidney and other cells with various pharmacological compounds. In a clinical setting, AMPK activity is enhanced with biguanides, including the antidiabetic drug metformin. The natural stilbenoid resveratrol can also be effective to treat patients with type 2 diabetes and associated complications, such as diabetic nephropathy (Kim et al., 2013; Kitada et al., 2011; Wang et al., 2017). Metformin, resveratrol and the AMPK activator AICAR (5-aminoimidazole-4-carboxamide 1-*β*-D-ribofuranoside) continue to be evaluated in clinical trials (https://clinicaltrials.gov/ct2/home). At the bench, AICAR, the biguanide phenformin, resveratrol and the ATP-competitive inhibitor compound C have been important tools to define the functions of AMPK (Kodiha, Ho-Wo-Cheong & Stochaj, 2011 and references therein). For example, these agents helped to uncover the mechanisms underlying mitochondrial biogenesis during long-term AMPK activation (Bhargava & Schnellmann, 2017).

As the main providers of cellular ATP, mitochondria are crucial for cell growth, proliferation and homeostasis. The major driving force for mitochondrial ATP production is the electrochemical gradient across the inner mitochondrial membrane (Bullon, Marin-Aguilar & Roman-Malo, 2016). The mitochondrial membrane potential (Δ*ψ*_m_) can hyperpolarize, diminish or collapse in response to stress, aging, and pathological or environmental cell insults (Srinivasan et al., 2017). Under these conditions, the quantitative assessment of the mitochondrial membrane potential provides an important approach to measure the impact on the organelle. Such measurements can be performed with several MitoTracker dyes. Relevant to our study, MitoTracker^®^ CMX ROS is a fixable fluorescent compound that generates information on the membrane potential across the inner mitochondrial membrane (Kodiha et al., 2015; Puleston, 2015 and references therein).

Several studies have shown that the properties of mitochondria vary widely between different tissues. Moreover, even within the same cell mitochondrial populations are heterogeneous (Collins et al., 2002; Kuznetsov & Margreiter, 2009; Kuznetsov et al., 2004; Woods, 2017). This heterogeneity includes morphological and functional differences between individual organelles. The functional diversity of mitochondria within a single cell may reflect their diverse contributions to physiological processes, such as metabolism, production of reactive oxygen species, calcium homeostasis, regulation of cell death and differentiation, as well as cell- and tissue-specific functions. To date, the signaling pathways that determine the heterogeneity of mitochondria are ill-defined (Kuznetsov & Margreiter, 2009; Woods, 2017).

The long-term impact of AMPK modulators on mitochondrial biogenesis is well established. By contrast, their immediate effects on mitochondria are poorly understood. To better characterize these processes, our current contribution evaluated the short-term changes of mitochondrial morphology and membrane potential elicited by commonly used pharmacological AMPK modulators. In addition, we have generated new information that relates to the heterogeneous properties of mitochondria in individual cells.

## Materials & Methods

### Growth of LLC-PK1 renal proximal tubule cells, incubation with pharmacological compounds and MitoTracker^®^ CMX ROS

LLC-PK1 and MCF7 cells were initially obtained from ATCC and grown as published (Kodiha et al., 2015). Cells were cultured in high glucose DMEM, containing 8% FBS, sodium pyruvate and antibiotics. Cells were treated and analyzed between passage 3 and 12 at a confluency of ∼70%.

**Figure 1:**
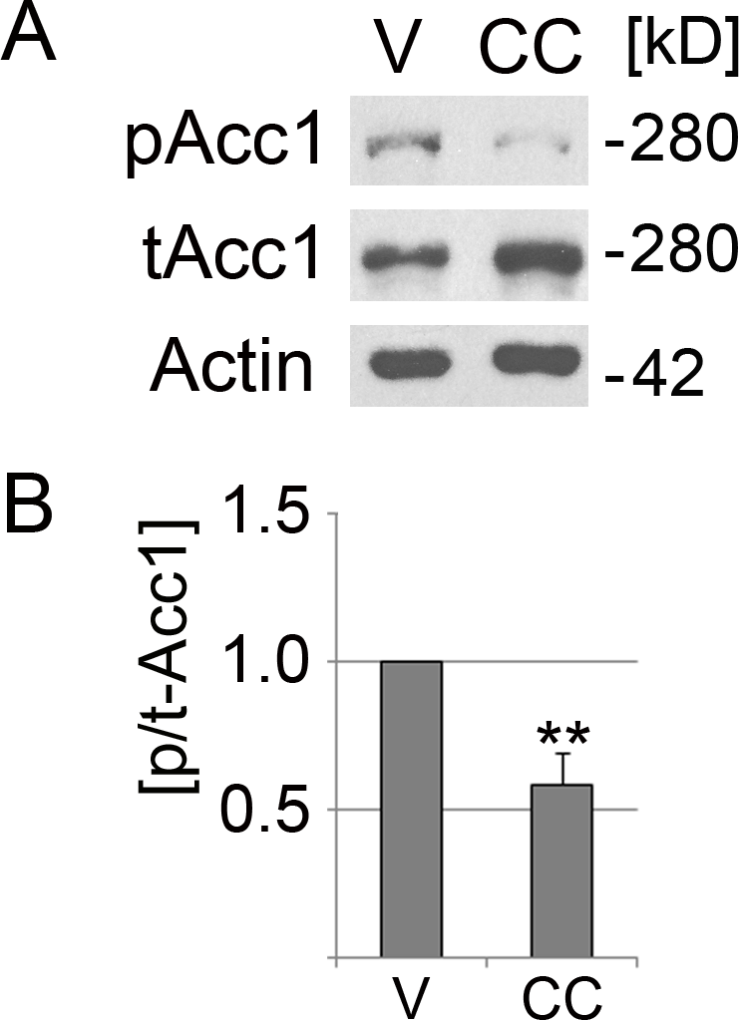
Compound C reduces significantly the phosphorylation of Acc1. LLC-PK1 cells were incubated with the vehicle DMSO (V) or compound C (CC) for 2 h at 37 °C. (A) Crude cell extracts were analyzed by Western blotting with antibodies against Acc1 phosphorylated on Ser79 (pAcc1) or total Acc1 (tAcc1). Actin was used as loading control. The molecular mass of Acc1 (280 kD) and the position of a 42 kD marker protein are indicated at the right margin. (B) The graph depicts the quantification of ECL signals. For each experiment, the ratio of ECL signals for pAcc1 and tAcc1 (p/t-Acc1) was determined. Data were normalized to the DMSO control (defined as 1.0 on the *y*-axis). Bars represent the average +SEM of four independent experiments. Student’s *t*-test demonstrates the significant reduction of the p/t Acc1 ratio upon treatment with compound C; **, *p* < 0.01.

AICAR (Cell Signaling Technology, #9944) was added as an aqueous solution. Phenformin (P7045; Sigma-Aldrich, St. Louis, MO, USA) and resveratrol (R5010; Sigma-Aldrich) were dissolved in DMSO; the solvent was present during the incubation period at a final concentration of 0.4%. For AMPK activation, LLC-PK1 cells were treated for 1 h at 37 °C with 5 mM phenformin, 200 µM resveratrol, or 1 mM AICAR. Controls were incubated with the vehicle DMSO or water, as appropriate. Compound C (Calbiochem, #171260; MilliporeSigma, Burlington, MA, USA) serves as an ATP-competitive inhibitor of AMPK; the agent was added for 2 h at a final concentration of 40 µM in 0.4% DMSO (vol/vol). This inhibitor concentration has previously been used for different mammalian cells (Benziane et al., 2009; Kim et al., 2008). Under these conditions, Acc1 phosphorylation on Ser79 was significantly reduced in LLC-PK1 cells (Fig. 1).

To determine the impact of different AMPK modulators on the mitochondrial membrane potential, cells grown on polylysine coated coverslips were treated with the pharmacological compound or vehicle, followed by incubation with MitoTracker^®^ CMX ROS (Life technologies, M7512). MitoTracker^®^ CMX ROS was added as a 1:5,000 dilution in DMEM. After 30 min at 37 °C, cells were fixed for immunostaining.

### Immunofluorescence, image acquisition and analysis

Fixed cells were processed for immunofluorescence following published procedures (Kodiha et al., 2015). Specifically, upon incubation with MitoTracker^®^ CMX ROS, culture medium was removed and coverslips were washed once with pre-warmed PBS. All subsequent steps were carried out at room temperature. Cells were fixed with 3.7% formaldehyde/PBS for 15 min and rinsed with PBS. After membrane permeabilization with 0.1% Triton X-100/2 mg/ml BSA/0.1% NaN_3_ for 10 min, non-specific binding sites were blocked for 1 h with 0.05% Tween 20, 5% fetal bovine serum, 1 mM NaN_3_ (blocking buffer). Incubation with primary antibodies against Tom70 (Fan et al., 2011; diluted 1:250 in blocking buffer) was overnight. Samples were washed three times for 10 min with blocking buffer and incubated with affinity-purified secondary antibodies (Alexa^®^488-conjugated anti-rabbit IgG, #711-545-152; Jackson ImmunoResearch, West Grove, PA, USA). After 2 h, coverslips were washed with blocking buffer (three times, 10 min for each step). DNA was stained with 1 µg/ml 4′,6-diamidino-2-phenylindole (DAPI) in blocking buffer and coverslips were mounted onto slides.

Images were acquired with a Zeiss LSM510 confocal microscope, using a 63 × oil immersion objective (*NA* = 1.4). Acquisition was with four-line averaging and a pixel time of 1.6 µs. Quantification of fluorescence signals was carried out with MetaXpress software. Tom70 provided a marker to identify mitochondrial compartments. Prior to the quantification of fluorescence intensities, background signals were subtracted for each image. Tom70 and MitoTracker^®^ CMX ROS pixel intensities were then measured for the compartments demarcated with Tom70. As described by us (Kodiha et al., 2015) and others (Higuchi-Sanabria et al., 2016), signals obtained for Tom70 were used as indicators of mitochondrial mass. The mitochondrial function was evaluated as the MitoTracker/Tom70 ratio for mitochondrial compartments. MitoTracker/area represented the MitoTracker^®^ CMX ROS fluorescence intensity divided by the mitochondrial area; the mitochondrial area was defined with Tom70. Tom70/area represents the pixel intensity for Tom70 divided by the mitochondrial area.

To characterize perinuclear and peripheral mitochondria, nuclei were identified with DAPI, and regions-of-interest were selected as concentric rings surrounding the nucleus. Each ring had a width of 1 µm; there was no overlap between consecutive rings. See ‘Results’ and ‘Discussion’ for details.

### Western blotting

Upon incubation with vehicle or AMPK modulator, LLC-PK1 cells were washed twice in PBS and stored at −70 °C. Processing for Western blotting was described previously (Kodiha, Ho-Wo-Cheong & Stochaj, 2011). One-fold concentrated gel sample buffer contained the following inhibitors of proteases and phosphatases: 1 mM phenylmethylsulfonyl fluoride, EDTA-free complete protease inhibitor cocktail (Roche), 20 mM *β*-glycerophosphate, 1 mM NaN_3_, 2.5 mM NaF). In brief, samples were resuspended in 0.5-fold concentrated gel sample buffer pH 8, incubated for 15 min at 95 °C and vortexed 3 min with glass beads. Insoluble material was removed by centrifugation (5 min, 13,000 rpm, microfuge). To precipitate proteins, supernatants were incubated with 10% TCA (final concentration, weight/volume) for 20 min on ice. Sediments were collected (2 min, 13,000 rpm, microfuge) and resuspended in 2-fold concentrated gel sample buffer, pH 8, containing protease and phosphatase inhibitors (Kodiha et al., 2007). Aliquots were separated by SDS-PAGE on gradient polyacrylamide gels. Samples were first assessed by staining of nitrocellulose filters with Ponceau S and immunoblotting with antibodies against actin. Based on these initial results, the volume of samples was adjusted for subsequent blots to achieve comparable loading.

Antibodies for Western blotting were purchased from the suppliers indicated and diluted as follows: Acc1 phosphorylated on Ser79 (#3661; 1:1,500; Cell Signaling Technology, Danvers, MA, USA), total Acc1 (Cell Signaling Technology, #3662; 1:1,500), affinity-purified antibodies against Tom70 (1:1,500), ERK1/2 dually phosphorylated on Thr202/Tyr204 (Cell Signaling Technology, #9106; 1:2,000), total ERK1/2 (Cell Signaling Technology, #4695; 1:2,000) and actin (1:100,000; Chemicon, MilliporeSigma, Burlington, MA, USA). Affinity-purified HRP-conjugated secondary antibodies (Jackson ImmunoResearch, 1:2,000) were used for the detection of bound primary antibodies by enhanced chemiluminescence (ECL). Multiple exposures were captured on film (M Plus Half Blue; Mandel Scientific Inc., Guelph, Canada) to prevent saturation of the signal.

### Statistics

Graphs were produced with Microsoft Excel software; they depict results as means +SEM. Statistical evaluation was performed by Student’s *t*-test (two-tailed) or One-way ANOVA, combined with Bonferroni correction. Details of the statistical assessment are provided in the figure legends. A *p*-value < 0.05 was considered significant.

## Results

### Pharmacological AMPK modulators affect MitoTracker^®^ CMX ROS accumulation in mitochondria

The mechanisms of action have been discussed previously for AMPK activators (Guigas & Viollet, 2016; Hardie, 2018; Hawley et al., 2010; Kodiha, Ho-Wo-Cheong & Stochaj, 2011). In brief, phenformin and resveratrol diminish mitochondrial ATP production, thereby decreasing the AMP/ATP ratio and activating AMPK. Resveratrol can also reduce the abundance of the phosphatases PP2A and PP2C (Li, Li & Ren, 2009); both enzymes dephosphorylate Thr183/Thr172 of the *α*-subunit. AICAR activates AMPK by generating an AMP mimetic. Compound C inhibits AMPK in LLC-PK1 cells; the agent decreased significantly the phosphorylation of Acc1 in mammalian cells (Fig. 1 and publications by others: Peng et al., 2012; Wang et al., 2013).

Tom70 resides in the outer mitochondrial membrane and is an essential component of the mitochondrial protein import machinery (reviewed in Wenz et al., 2015). In the current study, Tom70 provided an established marker to demarcate mitochondria. Specifically, Tom70 defined the mitochondrial area and informed on the mitochondrial mass (Higuchi-Sanabria et al., 2016; Kodiha et al., 2015).

Using confocal microscopy and quantitative image analysis, we measured changes in MitoTracker^®^ CMX ROS (in the following referred to as MitoTracker) fluorescence under conditions known to activate or inhibit AMPK in renal proximal tubule cells (Kodiha, Ho-Wo-Cheong & Stochaj, 2011 and Fig. 1). To this end, we evaluated the short-term effects of compound C, AICAR, phenformin and resveratrol on mitochondria in cell culture models. These effects are not well characterized for renal proximal tubule cells, but clinically relevant to different types of kidney injury (Morigi et al., 2015; Nowak, Bakajsova & Samarel, 2011). Our focus was on the mitochondrial membrane potential, because it is critical for multiple aspects of mitochondrial function, as exemplified by ATP production and the import of nuclear-encoded proteins into the mitochondrial matrix (Wenz et al., 2015).

**Figure 2:**
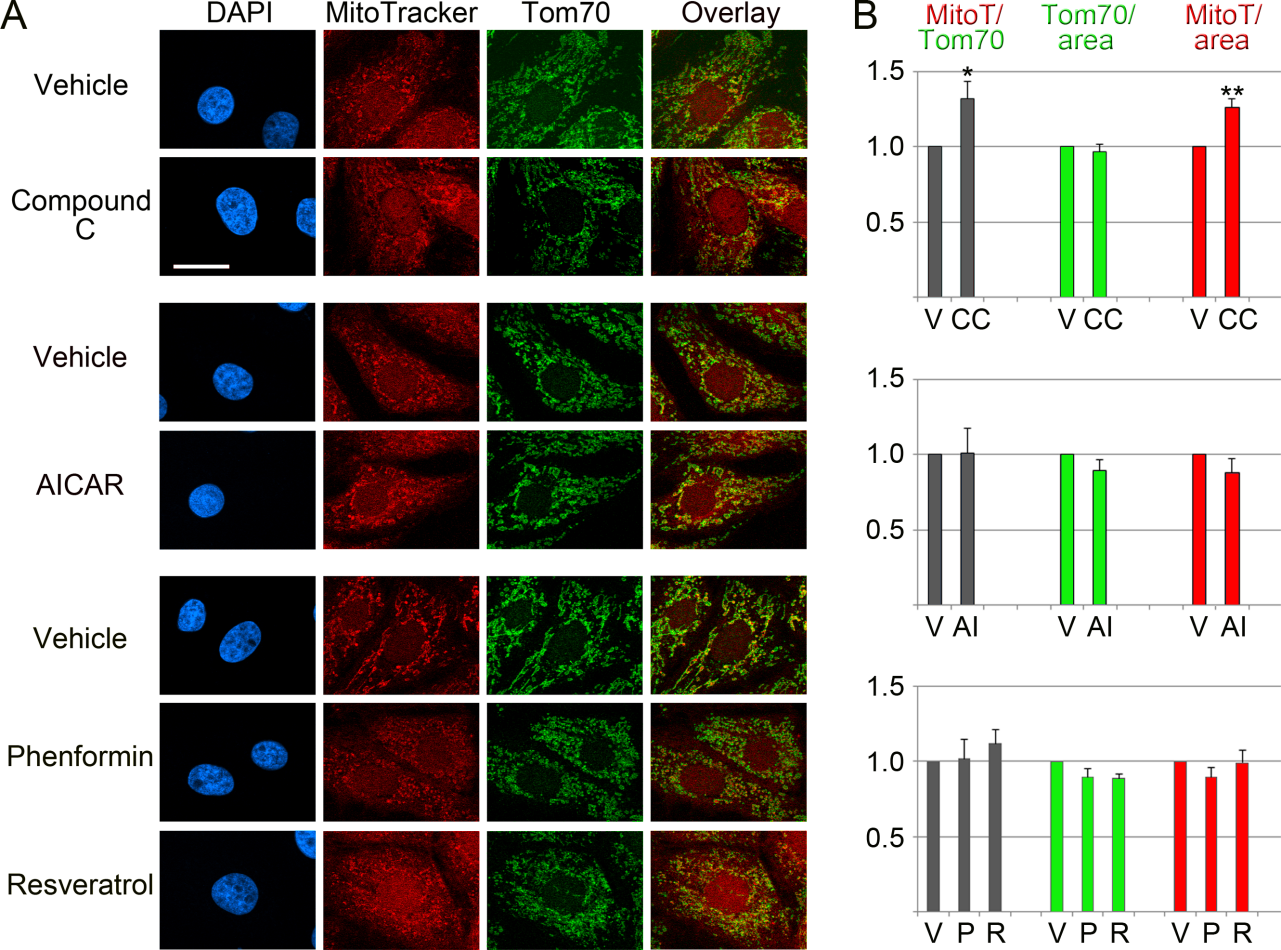
Effects of pharmacological AMPK modulators on mitochondria. LLC-PK1 cells were incubated with vehicle, compound C, AICAR, phenformin or resveratrol. (A) MitoTracker staining and the detection of Tom70 were carried out as described in ‘Materials & Methods’. DAPI staining visualized the nuclei. Scale bar is 20 µm. (B) Quantification of fluorescence signals. Results are shown for three to four independent experiments; at least 35 cells were analyzed for each data point and experiment. Data were normalized to vehicle controls. *Y*-axes display arbitrary units for MitoTracker/Tom70, Tom70/mitochondrial area, MitoTracker/mitochondrial area; bars depict averages +SEM. V, vehicle; CC, compound C, AI, AICAR; P, phenformin; R, resveratrol. Student’s *t*-test was performed to compare compound C to the vehicle DMSO, or AICAR to the vehicle water. One-way ANOVA combined with Bonferroni post-hoc analysis identified significant differences between the vehicle DMSO and samples treated with phenformin or resveratrol; *, *p* < 0.05; **, *p* < 0.01.

Figure 2 shows that compound C induced acute changes for several mitochondrial parameters. Thus, compound C increased significantly (+32%) the ratio of MitoTracker/Tom70 signals and the MitoTracker pixel intensity/mitochondrial area (+26%). By contrast, there were no profound effects on the relative abundance of Tom70 (Tom70/mitochondrial area in Fig. 2). Unlike compound C, the AMPK activators AICAR, phenformin and resveratrol did not cause significant changes for the mitochondrial parameters evaluated by us (Fig. 2), although they efficiently activated AMPK in LLC-PK1 cells (Kodiha, Ho-Wo-Cheong & Stochaj, 2011).

Together, these results demonstrate that the short-term treatment with compound C, which is accompanied by AMPK inhibition, has significant impact on the mitochondrial membrane potential. Given that mitochondria participate in a multitude of cellular processes, this is important information relevant to the physiological changes elicited by compound C.

### Impact of AMPK modulators on Tom70 abundance

**Figure 3:**
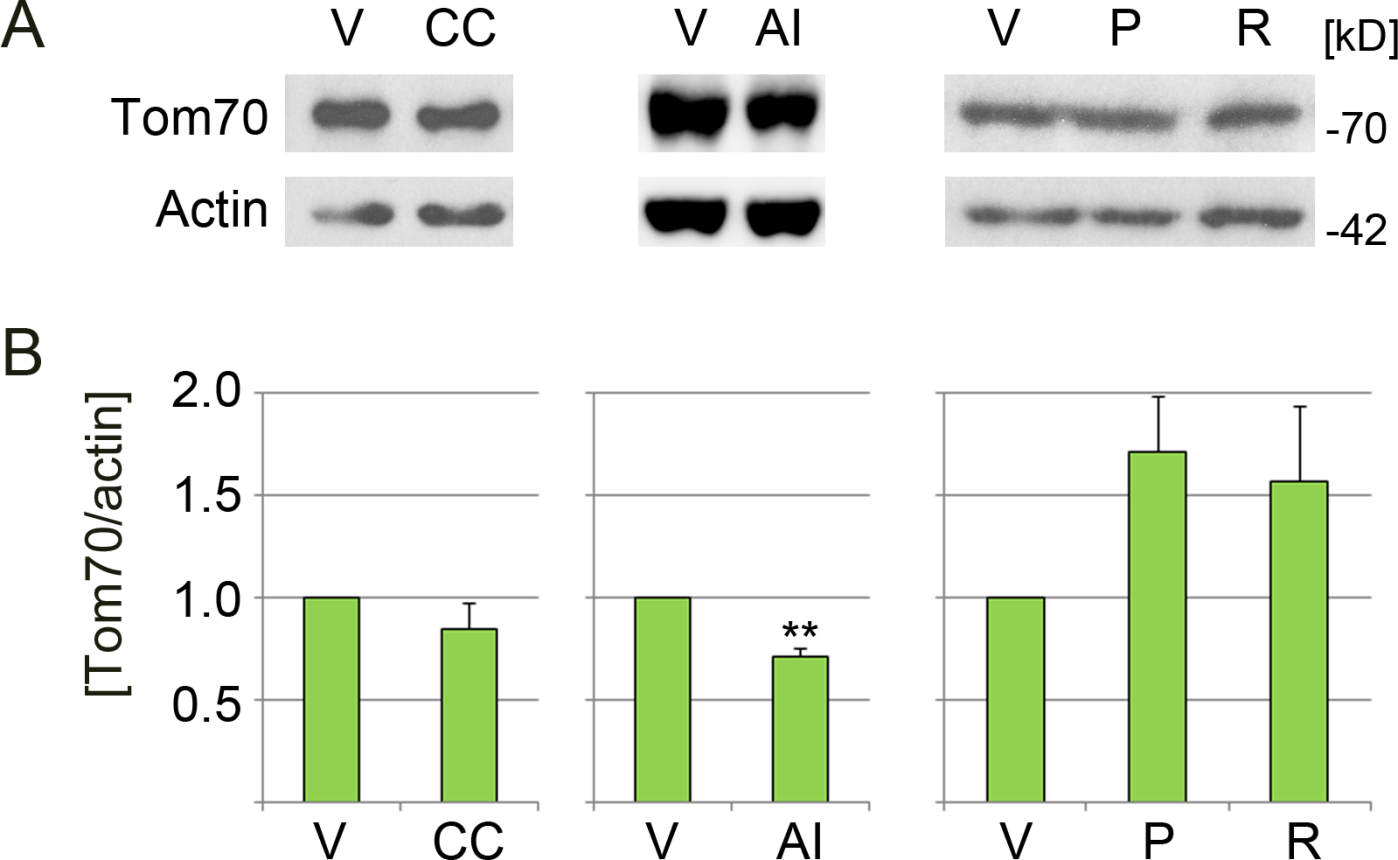
Effect of pharmacological AMPK modulators on the abundance of Tom70. LLC-PK1 cells were treated with vehicle (V), compound C (CC), AICAR (AI), phenformin (P) or resveratrol (R). (A) Crude extracts were examined by Western blotting for the abundance of Tom70, actin served as loading control. Molecular masses of marker proteins in kD are indicated at the right margin. (B) Bars represent the average of results +SEM for at least three independent experiments. The *y*-axis shows values for the Tom70/actin ratio; data for each individual experiment were normalized to the vehicle control. Student’s *t*-test (DMSO vs. compound C; water vs. AICAR) or One-way ANOVA combined with Bonferroni posthoc analysis (DMSO, phenformin, resveratrol) was used to identify significant differences; **, *p* < 0.01.

Western blot analyses were conducted to identify possible drug-induced changes in Tom70 protein concentration. Figure 3 depicts a reduction of Tom70 upon incubation with compound C (diminished by 15%) or AICAR (by 29%). By contrast, Tom70 abundance increased with phenformin (+71%) and resveratrol (+57%), but the extent was variable between experiments. Since Tom70 is important for the import of mitochondrial matrix proteins with internal targeting sequences, it is possible that phenformin and resveratrol rapidly enhance mitochondrial protein import. Long-term, this could contribute to the AMPK-dependent biogenesis of mitochondria, an established outcome of AMPK activation.

Data in Fig. 2 demonstrate that compound C changed mitochondrial properties. In particular, the reagent elevated MitoTracker signals relative to Tom70 by 32%. Different scenarios may explain these results: first, compound C leads to mitochondrial hyperpolarization. Second, the agent causes a loss of Tom70. Third, compound C promotes a combination of these events.

As shown in Figs. 2 and 3, compound C did not drastically diminish the association of Tom70 with mitochondria or overall Tom70 abundance (reduction by 15%, relative to the reference actin). At the same time, the MitoTracker/area signals increased (Fig. 2). Therefore, we favor the idea that compound C elevated the mitochondrial membrane potential. However, we do not rule out that additional mechanisms contributed to the effects of compound C.

### Mitochondria reorganize in response to compound C treatment

**Figure 4:**
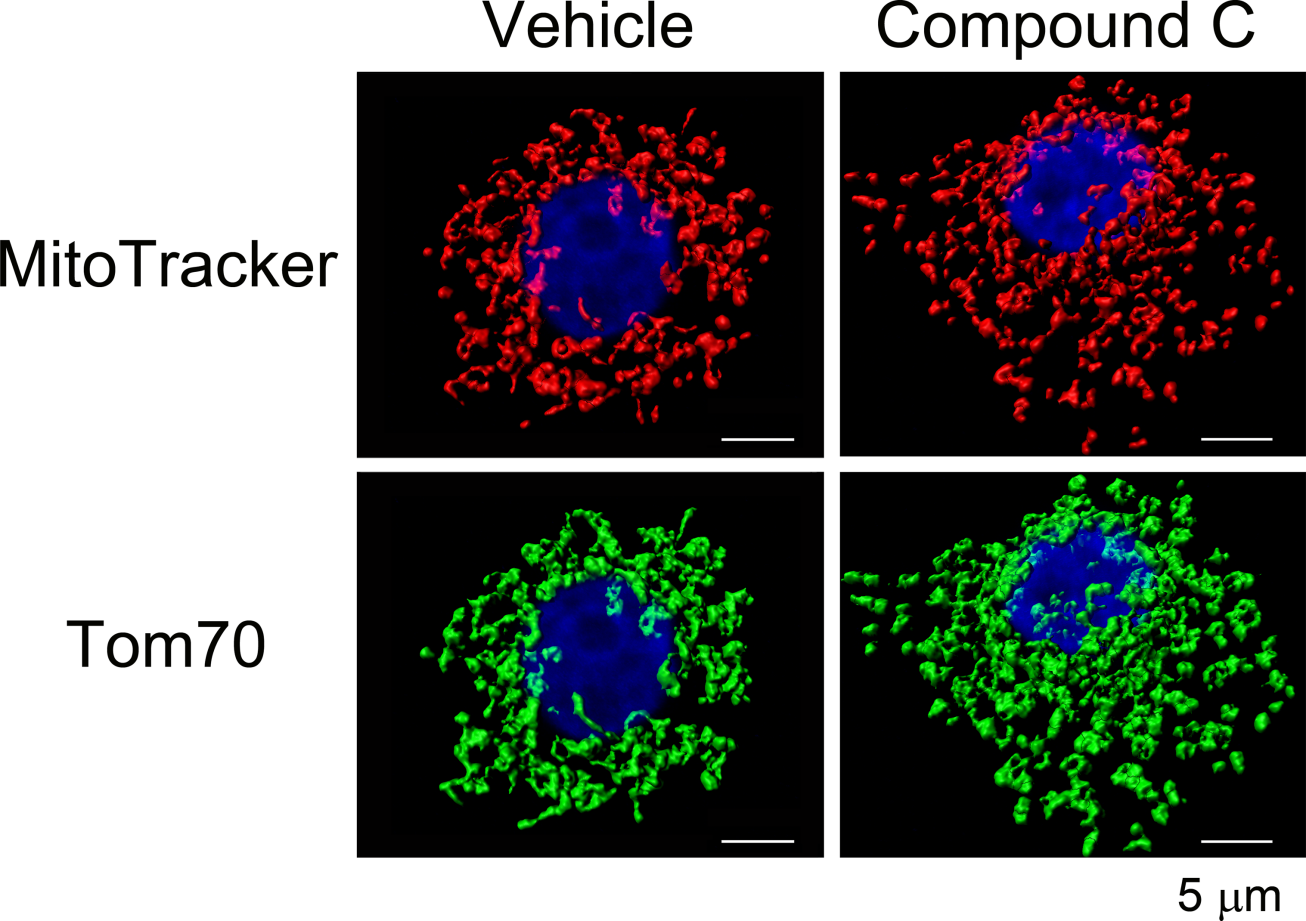
Impact of compound C on mitochondrial morphology. LLC-PK1 cells were treated with the vehicle DMSO or compound C and processed as described for Fig. 2. Mitochondrial morphology was visualized by 3D reconstruction for confocal stacks. Note that mitochondrial fragmentation with compound C was obvious for compartment demarcation with MitoTracker or Tom70. Scale bars for all panels are 5 µm.

Mitochondrial functions and morphology are closely linked; mitochondrial stress and other physiological events can promote organelle fragmentation (Srinivasan et al., 2017). Given the significant increase in the mitochondrial membrane potential elicited by compound C, we examined its possible impact on mitochondrial morphology. To this end, confocal image stacks were acquired for 3D image reconstruction. Based on MitoTracker fluorescence, Fig. 4 suggests a marked mitochondrial reorganization when cells were treated with compound C. This mitochondrial fragmentation was confirmed when mitochondria were visualized with the mitochondrial marker protein Tom70.

### Compound C stimulates dual ERK1/2 phosphorylation

**Figure 5:**
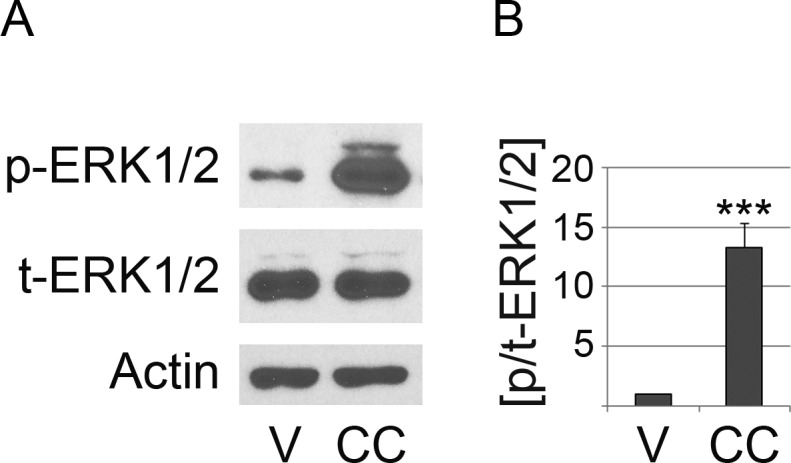
Compound C promotes the dual phosphorylation of ERK1/2 in renal proximal tubule cells. LLC-PK1 cells were incubated with vehicle (V) or compound C (CC) as described for Fig. 2. (A) Crude extracts were assessed for the dual phosphorylation of ERK1/2 on Thr202/Tyr204 (p-ERK1/2). The assessment of total ERK1/2 (t-ERK1/2) and the loading control actin are shown for comparison. (B) The ratio dual phosphorylated/total ERK1/2 (p/t-ERK1/2) was quantified for four independent experiments. Results were normalized to vehicle controls. Units on the *y*-axis represent the p/t-ERK1/2 ratio; bars depict the average +SEM. Student’s *t*-test identified significant differences between vehicle- and compound C-treated samples; ***, *p* < 0.001.

The hyperpolarization of mitochondria by compound C was surprising. One possible explanation was the potential crosstalk with other signaling pathways. As crosstalk between AMPK and ERK1/2 pathways has been reported (Tang et al., 2015), and ERK1/2 signaling has been linked to hyperpolarization (Nowak, 2002), we determined the effects of compound C on ERK1/2 activation. As shown in Fig. 5, treatment with compound C elicited an >10-fold increase in dual ERK1/2 phosphorylation on Thr202/Tyr204, an indicator of ERK1/2 activation. Notably, ERK1/2 stimulates mitochondrial fission (Cai et al., 2016). We propose that the mitochondrial fragmentation observed with compound C (Fig. 4) may be explained by ERK1/2 activation.

**Figure 6:**
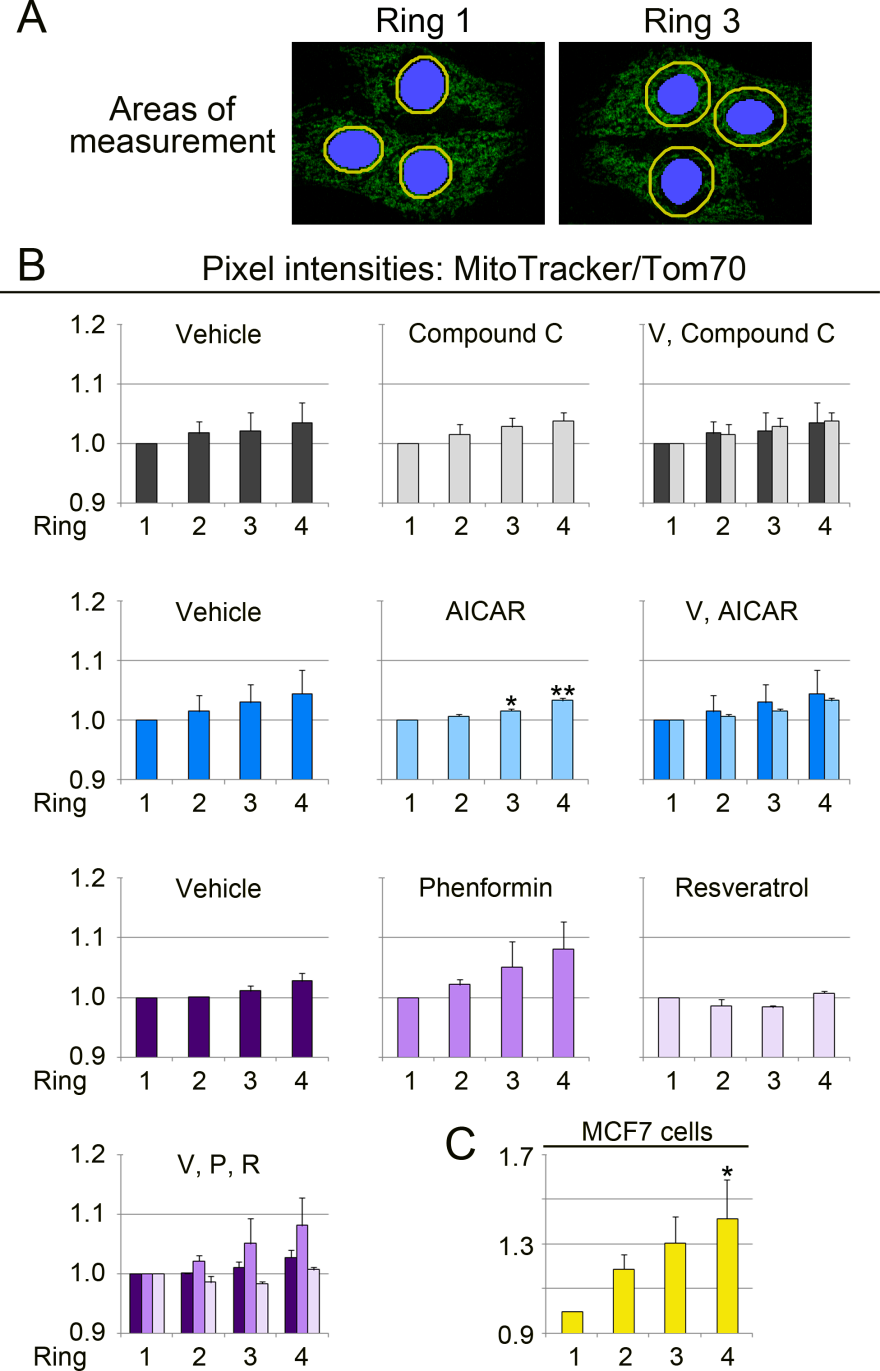
Quantitative image analysis links the MitoTracker/Tom70 ratio to the subcellular organelle location. (A) Image analysis method. Mitochondria were evaluated in regions representing adjacent, but non-overlapping concentric rings (yellow) that surround the nucleus (blue). The width of each ring was 1 µm. Four rings were assessed for each individual cell. Ring 1 and 3 are shown to illustrate the principle. (B) Measurement of the MitoTracker/Tom70 quotient in different regions of the cell. The MitoTracker/Tom ratio was calculated for LLC-PK1 renal proximal tubule cells. Results are depicted for two independent experiments. Data for *each* graph were normalized to ring 1, which represents the perinuclear region. The normalization enables a direct comparison of compound-induced changes in each ring-shaped region. It should be noted that Figs. 6–8 do not illustrate the overall drug-dependent effects that are shown in Fig. 2. *Y*-axes display the ratio MitoTracker/Tom70. Samples incubated with vehicle (V), compound C, AICAR, phenformin (P) or resveratrol (R) were examined. Each experiment evaluated between 34 and 51 cells for every condition. To simplify comparisons, results for the same experimental group are also combined in the same graph. One-way ANOVA with Bonferroni correction identified significant differences between ring 1 and other rings for each treatment; *, *p* < 0.05; **, *p* < 0.01. Ring 1 provided the reference for statistical evaluation. (C) MitoTracker/Tom70 values were measured for untreated MCF7 cells. In total, 18 cells were evaluated. One-way ANOVA with Bonferroni correction uncovered significant differences for ring 4 when compared to ring 1.

### The subcellular mitochondrial localization determines the function of the organelle

Depending on the physiological conditions, mitochondria can change their subcellular distribution to engage in specific functions. For example, mitochondria located in the vicinity of the nucleus promote the communication between both organelles (Al-Mehdi et al., 2012). At the cell periphery, mitochondria may support the function of ion pumps and protect against environmental insults (Kuznetsov & Margreiter, 2009).

While the heterogeneity of mitochondria within single cells is well established (Kuznetsov & Margreiter, 2009; Woods, 2017), it is not understood whether mitochondrial properties are in general controlled by their distance from the nucleus. To address this point, we designed an unbiased and systematic approach, depicted in Fig. 6A. This strategy used the MitoTracker/Tom70 ratio as readout to assess mitochondrial function in relation to the organelle position. Our assay quantifies MitoTracker/Tom70 signals for regions-of-interest that are at a defined distance from the nucleus. Concentric rings surrounding the nucleus and 1 µm wide were delineated by the image analysis software (Fig. 6A, yellow rings). Ring 1 was immediately adjacent to the nucleus, followed by ring 2 and 3. Ring 4 had the greatest distance from the nucleus.

**Figure 7:**
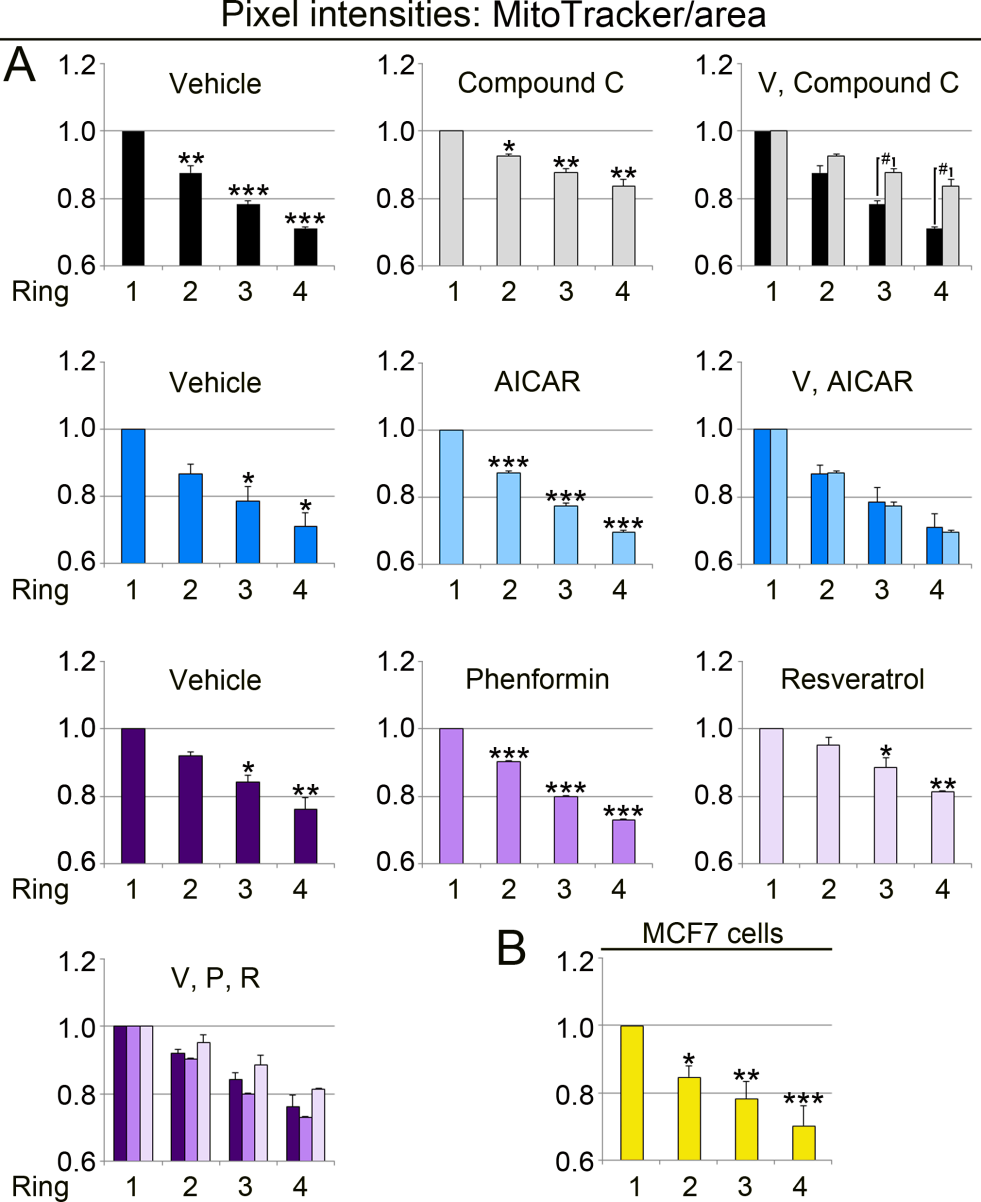
The subcellular position of mitochondria determines their MitoTracker/area value. The methods described in Fig. 6 assessed the MitoTracker/mitochondrial area quotient in ring-shaped regions surrounding the nucleus (see Fig. 6A). (A) Pixel intensities for MitoTracker/area were calculated for control and treated LLC-PK1 cells. *Y*-axes display the measurements for MitoTracker/area, normalized to ring 1. For statistical evaluation, ring 1 was compared to ring 2, 3 or 4 by One-way ANOVA with Bonferroni post-hoc analysis; *, *p* < 0.05; **, *p* < 0.01; ***, *p* < 0.001. In cells treated with compound C, rings 3 and 4 were significantly different from vehicle controls (Student’s *t*-test; #, *p* < 0.05). (B) MitoTracker/area values were determined for 18 untreated MCF7 cells. One-way ANOVA with Bonferroni post-hoc analysis showed that the MitoTracker/area quotient for ring 1 was significantly higher than for ring 2, 3 or 4; *, *p* < 0.05; **, *p* < 0.01; ***, *p* < 0.001.

**Figure 8:**
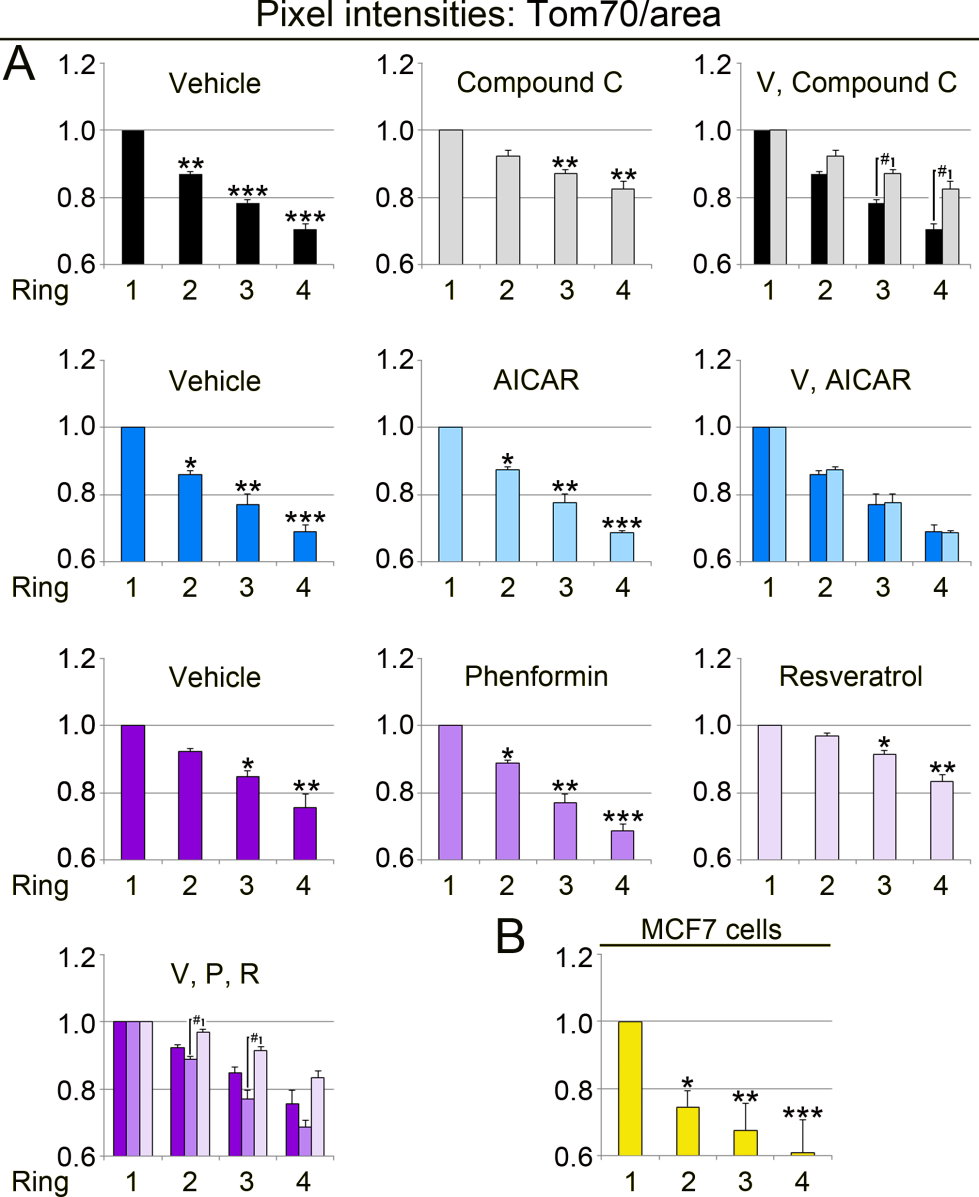
Quantitative image analyses demonstrate that the abundance of Tom70/mitochondrial area relies on the organelle position. (A) LLC-PK1 cells were examined for the subcellular distribution of Tom70. The pixel intensity for Tom70/mitochondrial area was calculated for each ring, as described for Fig. 6. *Y*-axes depict the ratio Tom70/mitochondrial area. For each data set, results were normalized to ring 1. One-way ANOVA combined with Bonferroni post-hoc analysis uncovered significant differences between ring 1 and ring 2, 3 or 4; *, *p* < 0.05; **, *p* < 0.01; ***, *p* < 0.001. Rings at the same position were further assessed in control and drug-treated cells. Top row: Student’s *t*-test compared rings for cells incubated with vehicle or compound C. Compound C increased significantly the signals for Tom70/area in rings 3 and 4; #, *p* < 0.05. Bottom row: Simultaneous comparison of all pairs by One-way ANOVA combined with Bonferroni correction revealed significant differences between phenformin (P) and resveratrol (R) for rings 2 and 3; #, *p* < 0.05. (B) In MCF7 cells, Tom70 abundance per mitochondrial area also depends on organelle location. Statistical assessment was performed by One-way ANOVA followed by Bonferroni post-hoc analysis. Compared to ring 1, values for Tom70/mitochondrial area were significantly lower in ring 2, 3 or 4; *, *p* < 0.05; **, *p* < 0.01; ***, *p* < 0.001. Note that for LLC-PK1 and MCF7 cells the ring-specific changes in the MitoTracker/area (Fig. 7) and Tom70/area (Fig. 8) quotients are not identical.

Experiments in Figs. 6–8 evaluated the *region-specific changes* in mitochondrial properties under different experimental conditions. To achieve this, each individual data set, either for vehicle or drug treatment, was normalized to ring 1. It should be noted that the experiments were not designed to assess the overall compound-induced changes of mitochondrial properties, as shown in Fig. 2.

Bar graphs in Figs. 6B and 6C show the results for our analyses. The MitoTracker/Tom70 values increased when perinuclear mitochondria were compared to organelles at the cell periphery. This was observed for vehicle controls and cells treated with different pharmacological agents. Our data suggest that mitochondrial function is affected by the organelle proximity to the nucleus. Interestingly, the location-dependent differences in mitochondrial properties were enhanced for each concentric area by phenformin, but diminished by resveratrol. By contrast, compound C and AICAR had only minor effects.

It was possible that the impact of subcellular location on mitochondrial properties was limited to renal proximal tubule cells. To test this hypothesis, we selected MCF7 human breast cancer cells as an additional model system. The rationale for this choice was as follows. First, MCF7 cells differ in origin from porcine LLC-PK1 cells, which were derived from the renal proximal epithelium. Second, we established earlier that the mitochondria in MCF7 cells can be assessed with the protocols developed by us (Kodiha et al., 2015). Applying these protocols, Fig. 6C demonstrates that the spatially controlled heterogeneity of mitochondria was not restricted to the kidney proximal tubule, but was preserved in other cell types.

Data in Fig. 6 did not reveal whether the region-specific differences were caused by changes in the MitoTracker signal, Tom70 or both. To define the contribution of both variables, we calculated the values for MitoTracker/mitochondrial area (Fig. 7, MitoTracker/area) and Tom70/mitochondrial area (Fig. 8, Tom70/area). Interestingly, the MitoTracker/area and Tom70/area quotient declined from the nucleus towards the cell periphery. For all conditions examined, the mitochondrial properties in ring 3 and 4 were significantly different from ring 1, which is perinuclear. We also demonstrated that compound C significantly increased the values for MitoTracker/area and Tom70/area in ring 3 and 4 as compared to the vehicle control (Figs. 7 and 8). In addition, phenformin and resveratrol had distinct region-dependent effects on mitochondria. These differences between phenformin and resveratrol were significant for Tom70/area in ring 2 and 3 (Fig. 8).

Collectively, our data uncovered functional divergence of mitochondria that are determined by the cellular position of the organelle relative to the nucleus. We also showed that both MitoTracker and Tom70 signals per mitochondrial area are dependent on the subcellular organelle location. Interestingly, the extent of region-specific changes was different for MitoTracker/area and Tom70/area.

## Discussion

Our results provide new knowledge on the physiological changes that AMPK modulators elicit in cultured mammalian cells. Our earlier work quantified the effects of AICAR, phenformin and resveratrol on AMPK *α*-subunit phosphorylation and the modification of its downstream target Acc1 in LLC-PK1 cells. Specifically, we have shown that these compounds are efficient AMPK activators using the conditions applied in the current study (Kodiha, Ho-Wo-Cheong & Stochaj, 2011), while compound C reduced the phosphorylation of Acc1 on Ser79 (Fig. 1). All of these pharmacological reagents are established tools that alter AMPK activation or enzymatic activity; they have been used widely for the analysis of AMPK and its physiological effects.

The current work is relevant, because we have focused on pharmacological tools that are commonly used in research or health applications. In particular, we revealed the short-term effects of AMPK modulators on mitochondrial characteristics; these effects were compound-specific.

The pharmacological agents evaluated by us impinged on the mitochondrial morphology, the membrane potential or Tom70, a key component of the mitochondrial protein import apparatus.

Strikingly, compound C caused not only mitochondrial hyperpolarization, but also mitochondrial fragmentation. These results are consistent with observations in other model systems. Thus, mitochondrial network fragmentation can occur under conditions that increase or diminish Δ*ψ*_m_ (De Vos et al. 2005; Galloway & Yoon, 2012, and references therein). In primary cultures of renal proximal tubule cells, activated PKC-ε elevated the mitochondrial membrane potential; this was accompanied by mitochondrial fragmentation (Nowak, Bakajsova & Samarel, 2011). Our work describes a similar scenario for compound C in LLC-PK1 cells. At present, it is not known whether PKC-ε plays a role for the mitochondrial changes elicited by compound C.

Some of the outcomes reported here are possibly caused by the crosstalk between AMPK and other signaling cascades, especially the ERK1/2 MAPK pathway. It is also conceivable that off-target effects contributed to the drug-induced mitochondrial changes. For instance, compound C inhibits the receptor tyrosine kinase VEGFR2 (also called FLK1) and ALK2 (Hao et al., 2010), a receptor for bone morphogenetic protein. However, under the conditions examined by us, VEGFR2 and ALK2 inhibition are likely to play only a minor role in LLC-PK1 cells. Although VEGFR2 is produced in kidney cells, the protein abundance is low in the renal proximal epithelium (Kanellis et al., 2000). At the same time, *ALK2* gene expression is low in the proximal tubule, at least in healthy kidneys (Leeuwis et al., 2011; Ten Dijke et al., 1993).

In addition to a better understanding of the short-term impact of pharmacological AMPK modulators, our study generated quantitative information on the heterogeneity of mitochondria in individual mammalian cells. In various cell types, mitochondrial organization ranges from randomly dispersed to highly connected networks (Kuznetsov & Margreiter, 2009). We have shown here that distinct mitochondrial subpopulations are present in LLC-PK1 and MCF7 cells. For both cell lines, the subcellular location, especially the distance from the nucleus, determined mitochondrial properties. This included multiple parameters: the MitoTracker/Tom70 ratio, MitoTracker signals/mitochondrial area, and Tom70 signals/mitochondrial area. Perinuclear mitochondria displayed a lower MitoTracker/Tom70 ratio when compared to organelles located at the cellular periphery. Therefore, our data suggest that mitochondria at the cell periphery are highly energized (Collins et al., 2002).

Interestingly, MitoTracker/area and Tom70/area values were particularly robust indicators of mitochondrial heterogeneity. We propose that both parameters will be useful to assess mitochondrial properties, including organelle heterogeneity, in various cell types and tissues. Since Tom70 is crucial for protein import into mitochondria, it will be interesting to examine the effects of subcellular location on the mitochondrial proteome.

The detection of mitochondrial subpopulations in renal proximal epithelial and MCF7 cells is reminiscent of the functional heterogeneity in several other cell types (reviewed in Kuznetsov & Margreiter, 2009). Interestingly, these subpopulations persisted when LLC-PK1 cells were treated with different agents. This may indicate that mitochondria have limited electrical connectivity, at least under normal growth conditions and during AMPK activation. Whether the connectivity is altered by compound C will have to be examined in the future.

To date, the physiological relevance of mitochondrial heterogeneity is beginning to emerge. For example, depending on the cytoplasmic location, the demands on mitochondrial performance can differ profoundly. Thus, highly energized mitochondria at the cell periphery sequester more Ca^2+^ ions than their perinuclear counterparts (Collins et al., 2002). Moreover, the cellular energy metabolism impinges on the morphology of mammalian mitochondria (reviewed in Westermann, 2012). It is also established that distinct cellular subpopulations of mitochondria differ in their responses to metabolites, stress and disease.

As proposed for other cell types (Kuznetsov & Margreiter, 2009), mitochondrial heterogeneity could contribute to a number of processes in renal proximal tubules. For example, ATP production in the vicinity of the nucleus could support energy-demanding processes, such as macromolecular trafficking through the nuclear pore (Kuznetsov & Margreiter, 2009) and the control of gene expression (Al-Mehdi et al., 2012). At the periphery of proximal tubule cells, mitochondria may support transporters in the plasma membrane, as exemplified by Na^+^/ K^+^- ATPase (Curthoys & Moe, 2014). Given the organization of polarized epithelial cells into apical and basolateral domains, it will be interesting to determine whether peripheral mitochondria display domain-specific properties.

Taken together, our current contribution uncovered two important aspects of mitochondrial biology that are amenable to pharmacological modulation: the MitoTracker/Tom70 ratio as readout for overall organelle function, and the region-specific mitochondrial heterogeneity. These insights set the stage to explore how AMPK impinges on mitochondrial properties in various cell types and tissues. Our work also provides new and unbiased imaging protocols to elucidate in a quantitative fashion the poorly defined mechanisms underlying the spatial organization and preservation of mitochondrial heterogeneity (Kuznetsov & Margreiter, 2009; Woods, 2017). Long-term, this information will be useful to alter rapidly local mitochondrial activities when cells are impaired by stress or disease conditions, such as acute kidney injury (Bhargava & Schnellmann, 2017; Eirin, Lerman & Lerman, 2017).

##  Supplemental Information

10.7717/peerj.5469/supp-1 Figure S1 Original blots related to Fig. 1. Original films for the blots depicted in Fig. 1 are shown. LLC-PK1 cells were incubated with the vehicle DMSO (V) or compound C (CC) as described in ‘Materials & Methods’. Crude extracts were prepared and evaluated by Western blotting with antibodies against Acc1 phosphorylated on Ser79 (pAcc1) or total Acc1 (tAcc1). The position of the 280kD band is indicated at the right margin.

10.7717/peerj.5469/supp-2 Supplemental Information 1 Statistical evaluations for different data sets Original results for Western blots and quantitative imaging are provided. Statistical evaluations for different data sets are shown.

10.7717/peerj.5469/supp-3 Figure S3 Original Western blots related to Fig. 3 LLC-PK1 cells were treated with vehicle (V), compound C (CC), AICAR (AI), phenformin (P) or resveratrol (R). Crude extracts were probed by Western blotting with antibodies against Tom70 and actin. The positions of Tom70 and actin are indicated for each data set. The molecular mass of marker proteins is depicted at the right margin of Western blots for the vehicle/compound C set. Different exposures of the films, as relevant to Fig. 3, are shown.

10.7717/peerj.5469/supp-4 Figure S5 Western blots related to Fig. 5 LLC-PK1 cells were treated with vehicle (V) or compound C (CC) as described in ‘Materials & Methods’. Crude cell extracts were probed with antibodies against ERK1/2 dually phosphorylated on Thr202/Tyr204 (p-ERK1/2), total ERK1/2 (tERK1/2) or actin. Molecular masses of marker proteins in kD are depicted at the left margin.

## Abbreviations

AI, AICAR: 5-Aminoimidazole-4-carboxamide 1-*β*-D-ribofuranoside
AMPK: 5′-AMP activated kinase
CC: compound C
DAPI: 4′,6-diamidino-2-phenylindole
Δ*ψ*_**m**_: mitochondrial membrane potential
MitoT: MitoTracker, MitoTracker^®^ CMX ROS
P: phenformin
R: resveratrol.

## Additional Information and Declarations

### Data Availability

The following information was supplied regarding data availability: The raw data are provided in the Supplemental Files.
