# Supplementary figures and images for: Defining the short-term effects of pharmacological 5′-AMP activated kinase modulators on mitochondrial polarization, morphology and heterogeneity
Source: PeerJ. 2018 Aug 30;6:e5469. doi: 10.7717/peerj.5469 (PMC6119600; doi:10.7717/peerj.5469)

# Western blots related to Fig.1

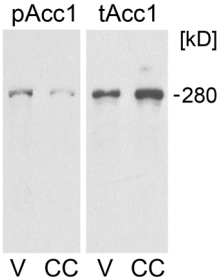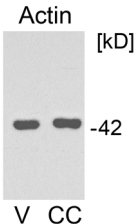

Supplement: Figure S1 — Original films for the blots depicted in Fig. 1 are shown. LLC-PK1 cells were incubated with the vehicle DMSO (V) or compound C (CC) as described in ‘Materials & Methods’. Crude extracts were prepared and evaluated by Western blotting with antibodies against Acc1 phosphorylated on Ser79 (pAcc1) or total Acc1 (tAcc1). The position of the 280kD band is indicated at the right margin. [file peerj-06-5469-s001.pdf]

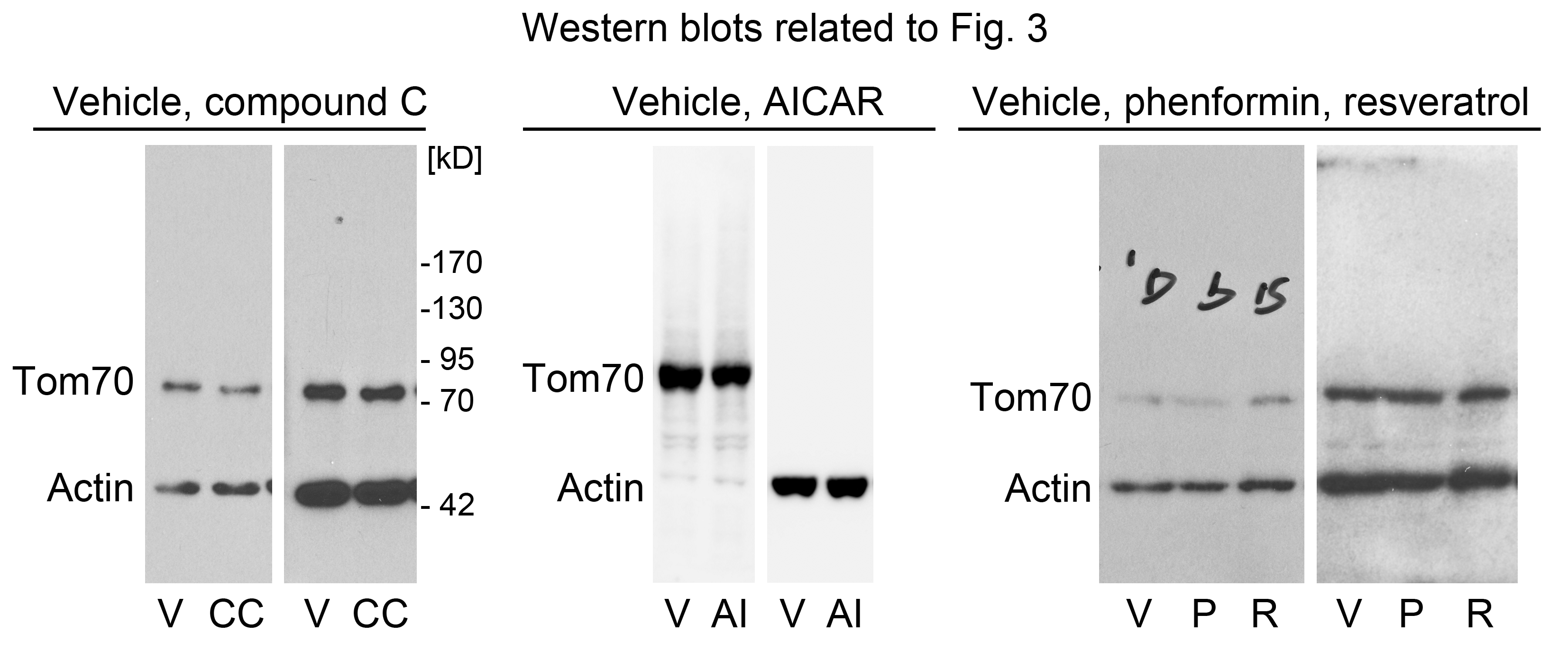

Supplement: Figure S3 — LLC-PK1 cells were treated with vehicle (V), compound C (CC), AICAR (AI), phenformin (P) or resveratrol (R). Crude extracts were probed by Western blotting with antibodies against Tom70 and actin. The positions of Tom70 and actin are indicated for each data set. The molecular mass of marker proteins is depicted at the right margin of Western blots for the vehicle/compound C set. Different exposures of the films, as relevant to Fig. 3, are shown. [file peerj-06-5469-s003.png]

# Western blots related to Fig. 5

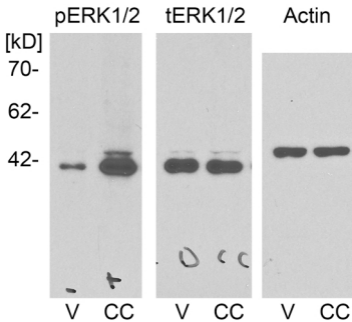

Supplement: Figure S5 — LLC-PK1 cells were treated with vehicle (V) or compound C (CC) as described in ‘Materials & Methods’. Crude cell extracts were probed with antibodies against ERK1/2 dually phosphorylated on Thr202/Tyr204 (p-ERK1/2), total ERK1/2 (tERK1/2) or actin. Molecular masses of marker proteins in kD are depicted at the left margin. [file peerj-06-5469-s004.pdf]
